# Triple Intergenotype Recombination of Human Astrovirus 5, Human Astrovirus 8, and Human Astrovirus 1 in the Open Reading Frame 1a, Open Reading Frame 1b, and Open Reading Frame 2 Regions of the Human Astrovirus Genome

**DOI:** 10.1128/spectrum.04888-22

**Published:** 2023-04-05

**Authors:** Hongyu Wei, Kattareeya Kumthip, Pattara Khamrin, Arpaporn Yodmeeklin, Nutthawadee Jampanil, Phitchakorn Phengma, Zhenfeng Xie, Nuthapong Ukarapol, Hiroshi Ushijima, Niwat Maneekarn

**Affiliations:** a Department of Microbiology, Faculty of Medicine, Chiang Mai University, Chiang Mai, Thailand; b Youjiang Medical University for Nationalities, Baise, Guangxi, China; c Center of Excellence in Emerging and Re-emerging Diarrheal Viruses, Chiang Mai University, Chiang Mai, Thailand; d Department of Pediatrics, Faculty of Medicine, Chiang Mai University, Chiang Mai, Thailand; e Division of Microbiology, Department of Pathology and Microbiology, Nihon University School of Medicine, Tokyo, Japan; City University of Hong Kong

**Keywords:** human astrovirus, gastroenteritis, intergenotype recombination, recombinant strain, Thailand

## Abstract

Human astrovirus (HAstV) strains exhibit high levels of genetic diversity, and many recombinant strains with different recombination patterns have been reported. The aims of the present study were to investigate the emergence of HAstV recombinant strains and to characterize the recombination patterns of the strains detected in pediatric patients admitted to the hospital with acute gastroenteritis in Chiang Mai, Thailand. A total of 92 archival HAstV strains detected in 2011 to 2020 were characterized regarding their open reading frame 1a (ORF1a) genotypes in comparison with their ORF1b genotypes to identify recombinant strains. The recombination breakpoints of the putative recombinant strains were determined by whole-genome sequencing and were analyzed by SimPlot and RDP software. Three HAstV strains (CMH-N178-12, CMH-S059-15, and CMH-S062-15) were found to be recombinant strains of three different HAstV genotypes, i.e., HAstV5, HAstV8, and HAstV1 within the ORF1a, ORF1b, and ORF2 regions, respectively. The CMH-N178-12 strain displayed recombination breakpoints at nucleotide positions 2681 and 4357 of ORF1a and ORF1b, respectively, whereas the other two recombinant strains, CMH-S059-15 and CMH-S062-15, displayed recombination breakpoints at nucleotide positions 2612 and 4357 of ORF1a and ORF1b, respectively. This is the first study to reveal nearly full-length genome sequences of HAstV recombinant strains with a novel recombination pattern of ORF1a-ORF1b-ORF2 genotypes. This finding may be useful as a guideline for identifying other recombinant HAstV strains in other geographical regions and may provide a better understanding of their genetic diversity, as well as basic knowledge regarding virus evolution.

**IMPORTANCE** Recombination is one of the mechanisms that plays a crucial role in the genetic diversity and evolution of HAstV. We wished to investigate the emergence of HAstV recombinant strains and to analyze the whole-genome sequences of the putative HAstV recombinant strains detected in pediatric patients with acute gastroenteritis in 2011 to 2020. We reported 3 novel intergenotype recombinant strains of HAstV5-HAstV8-HAstV1 at the ORF1a-ORF1b-ORF2 regions of the HAstV genome. The hot spots of recombination occur frequently near the ORF1a-ORF1b and ORF1b-ORF2 junctions of the HAstV genome. The findings indicate that intergenotype recombination of HAstV occurs frequently in nature. The emergence of a novel recombinant strain allows the new virus to adapt and successfully escape from the host immune system, eventually emerging as the predominant genotype to infect human populations that lack herd immunity against novel recombinant strains. The virus may cause an outbreak and needs to be monitored continually.

## INTRODUCTION

Human astrovirus (HAstV) infection is a common cause of acute gastroenteritis in children. HAstV is a nonenveloped, positive-sense, single-stranded RNA virus that belongs to the genus *Mamastroviridae* in the family *Astroviridae* (1). The HAstV genome of approximately 6,800 to 7,000 nucleotides consists of three open reading frames (ORFs), i.e., ORF1a, ORF1b, and ORF2 (2). Currently, HAstV has been classified into classic HAstV, novel HAstV-MLB, and novel HAstV-VA clades, with genotypes of HAstV1 to HAstV8, MLB1 to MLB3, and VA1 to VA5, respectively (1, 3). Genetic recombination is one of the mechanisms that plays an essential role in the genetic diversity and evolution of HAstV (1, 3, 4). To date, a number of intergenotype recombinant strains with ORF1b-ORF2 of HAstV genotypes, including HAstV1-HAstV2, HAstV1-HAstV3, HAstV1-HAstV4, HAstV1-HAstV5, HAstV2-HAstV5, HAstV2-HAstV8, HAstV3-HAstV2, HAstV3-HAstV5, and HAstV4-HAstV5, have been reported from several countries (5–12). Recently, we reported intergenotype recombinant strains with ORF1b-ORF2 of HAstV8-HAstV1, HAstV8-HAstV3, and HAstV3-HAstV2 genotypes among pediatric patients with acute gastroenteritis in Chiang Mai, Thailand (13). In addition, intergenotype recombinant strains with ORF1a-ORF2 of HAstV2-HAstV3, HAstV8-HAstV1, and HAstV8-HAstV2 (14) and HAstV4-HAstV5, HAstV7-HAstV2, and HAstV8-HAstV1 (15) have been reported. The aims of the present study were to investigate the emergence of recombinant strains and to characterize by whole-genome sequence analysis the recombination patterns within the ORF1a, ORF1b, and ORF2 regions of HAstV detected in children with acute gastroenteritis.

## RESULTS

### Determination of putative recombinant strains.

**FIG 1:**
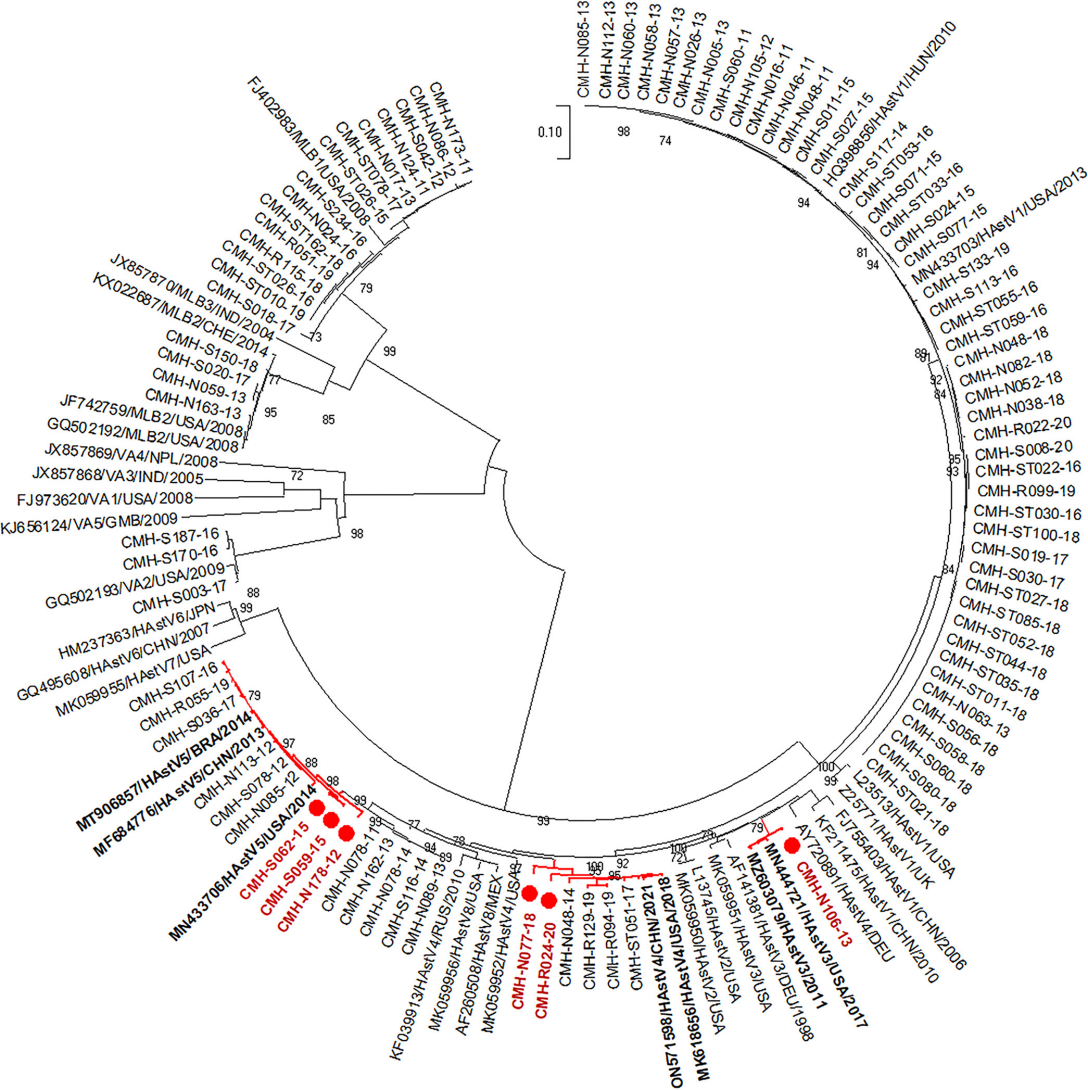
Phylogenetic analysis of the partial ORF1a region (663 bp) of 91 HAstV strains detected in Chiang Mai, Thailand, in 2011 to 2020. It should be noted that 1 HAstV strain (CMH-S015-20) was not included in this analysis because only a short ORF1a sequence could be obtained for that strain. The putative recombinant strains detected in this study are indicated by red circles. Astrovirus reference strains included in this analysis are indicated with their GenBank accession numbers and genotypes. HAstV, human astrovirus.

**FIG 2:**
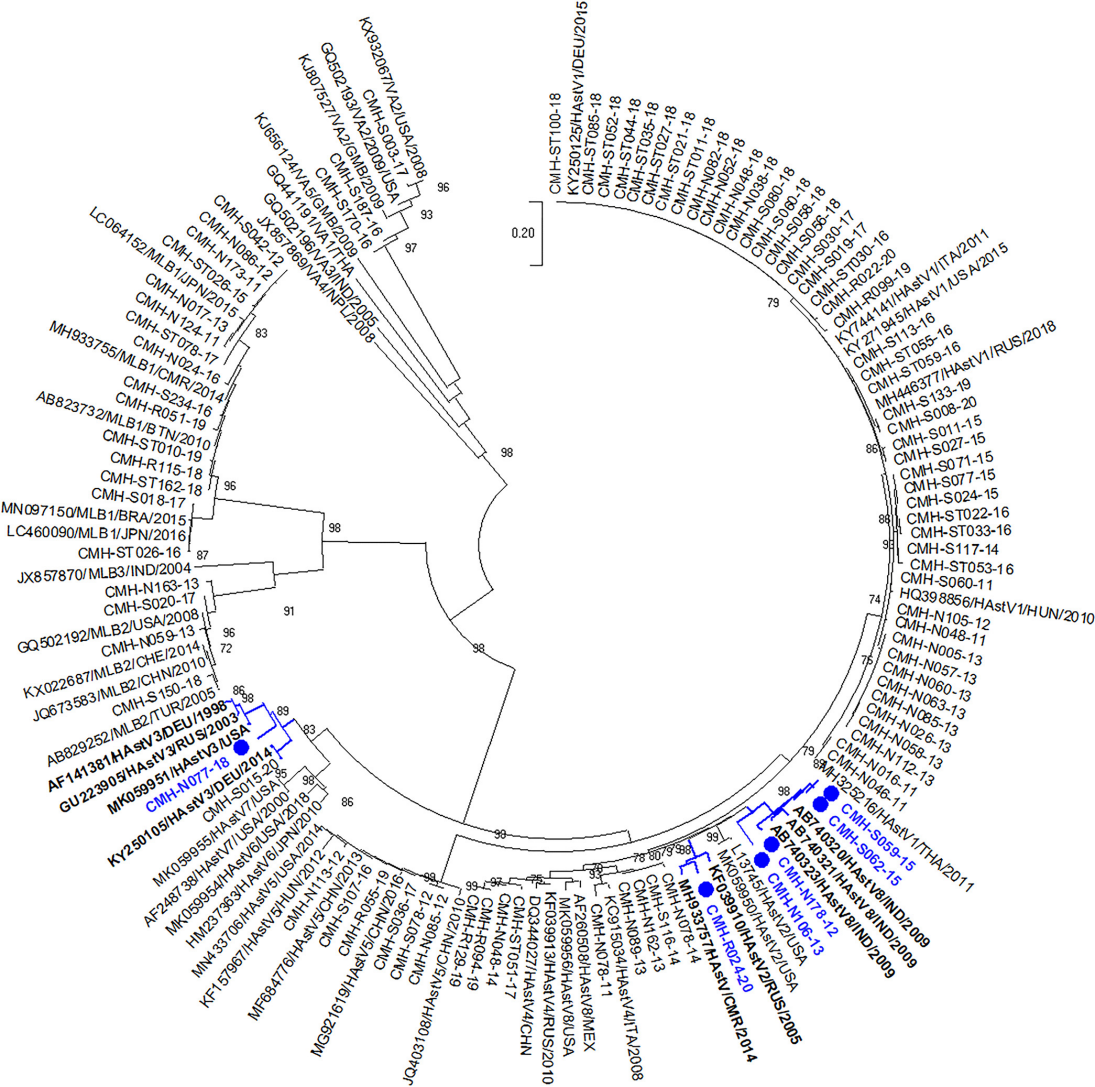
Phylogenetic analysis of the partial ORF1b region (326 bp) of 92 HAstV strains detected in Chiang Mai, Thailand, in 2011 to 2020. The putative recombinant strains detected in this study are indicated by blue circles. Astrovirus reference strains included in this analysis are indicated with their GenBank accession numbers and genotypes. HAstV, human astrovirus.

The RNA-dependent RNA polymerase (RdRp) genotypes of all 92 HAstV strains were identified by ORF1b nucleotide sequence analysis, as described in our previous studies (16, 17). To determine the putative recombinant strains, the assigned HAstV genotypes based on the partial ORF1a region were compared with their ORF1b genotypes. Phylogenetic analyses of the ORF1a and ORF1b regions of HAstV strains detected in this study are shown in Fig. 1 and 2, respectively. The results demonstrated that HAstV strains circulating in this area were relatively homogenous. The HAstV strains that were grouped into the same genotype for ORF1a and ORF1b showed nucleotide sequence identities among themselves ranging from 89.7 to 100% and from 89.4 to 100%, respectively. By comparing the virus genotypes assigned by ORF1a and ORF1b, a HAstV strain that had the same ORF1a and ORF1b genotypes was considered to be a nonrecombinant strain, whereas a virus that exhibited disagreement between the genotypes for ORF1a and ORF1b was considered to be a potential recombinant strain. Based on nonconcurrence between the ORF1a genotype and the ORF1b genotype, six HAstV strains were identified as putative recombinant strains. The CMH-N178-12, CMH-S059-15, and CMH-S062-15 strains were identified as HAstV5-HAstV8 for the ORF1a-ORF1b genotypes. The CMH-N106-13 strain was assigned as a HAstV3-HAstV8 recombinant strain, whereas CMH-N077-18 and CMH-R024-20 were defined as recombinant strains of HAstV4-HAstV3 and HAstV4-HAstV2, respectively. The remaining HAstV strains were nonrecombinant.

### Amplification of nearly full-length genomes of HAstV strains.

The nearly complete genomes of putative recombinant HAstV were successfully amplified from three strains, i.e., CMH-N178-12, CMH-S059-15, and CMH-S062-15, while the other three stains (CMH-N103-13, CMH-N077-18, and CMH-R024-20) failed to have their nearly complete genome sequences amplified and thus were excluded from the analysis. The nucleotide sequences obtained for the CMH-N178-12, CMH-S059-15, and CMH-S062-15 strains that had been successfully amplified were 6,607, 6,614, and 6,640 bp long, respectively. Based on the nucleotide positions in the full-length genome of HAstV1 (GenBank accession number HQ398856) used as a reference strain, the full-length genomes of CMH-N178-12, CMH-S059-15, and CMH-S062-15 were located at nucleotide positions 131 to 6737, 128 to 6741, and 134 to 6773, respectively. The genome sequence of each strain contained the sequences of the ORF1a, ORF1b, and ORF2 regions.

### Phylogenetic analysis of the ORF1a, ORF1b, and ORF2 regions.

**FIG 3:**
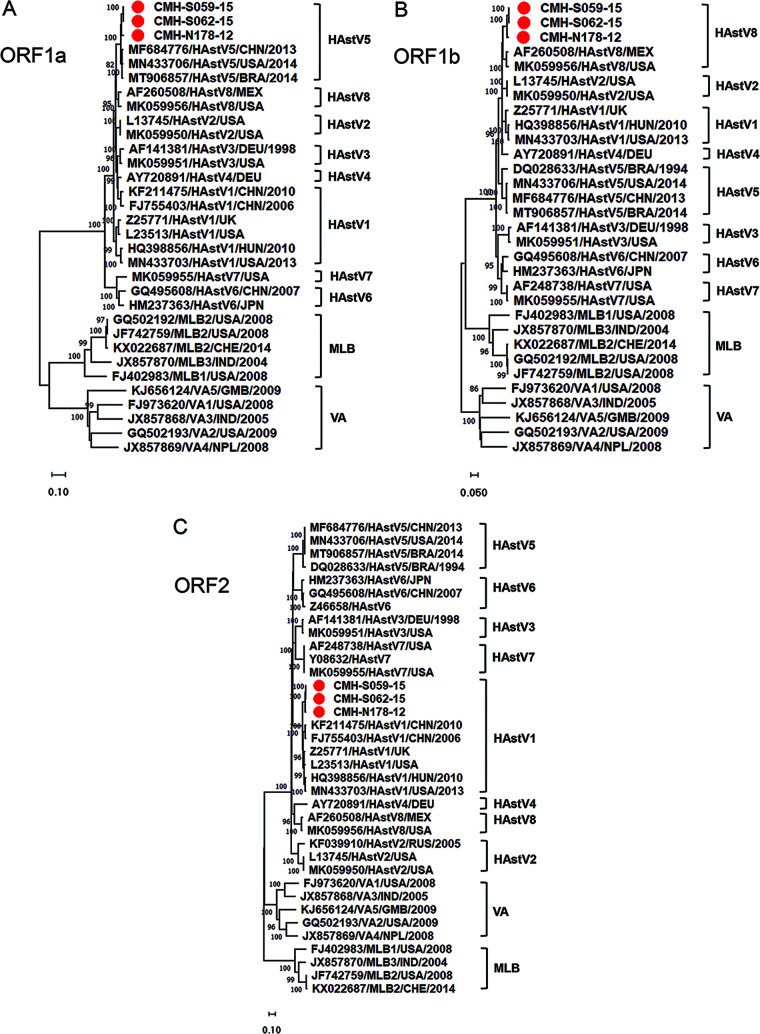
Phylogenetic analysis of HAstV recombinant strains based on nucleotide sequences of the ORF1a region (2,710 bp) (A), nucleotide sequences of the ORF1b region (1,540 bp) (B), and nucleotide sequences of the ORF2 region (2,350 bp) (C). The putative recombinant strains detected in this study are indicated by red circles. Astrovirus reference strains included in this analysis are indicated with their GenBank accession numbers and genotypes. HAstV, human astrovirus.

Phylogenetic trees for the nucleotide sequences of the ORF1a, ORF1b, and ORF2 regions of CMH-N178-12, CMH-S059-15, and CMH-S062-15 HAstV strains are shown in Fig. 3A, B, and C, respectively. In Fig. 3A, the phylogenetic tree for ORF1a of these strains (CMH-N178-12, CMH-S059-15, and CMH-S062-15) showed that they clustered closely among themselves, with nucleotide sequence identities ranging from 97.5 to 99.9%, and closely together with classic HAstV5 reference strains reported previously from China (GenBank accession number MF684776), Brazil (GenBank accession number MT906857), and the United States (GenBank accession number MN433706), with nucleotide sequence identities ranging from 96.4 to 97.5%. The data indicated that the ORF1a genotype of these three strains was a classic HAstV5 genotype. In Fig. 3B, the phylogenetic tree for ORF1b revealed that these strains also clustered closely together among themselves, with nucleotide sequence identities ranging from 97.8 to 99.9%, and clustered closely together with reference strains of the classic HAstV8 genotype reported previously from Mexico (GenBank accession number AF260508) and the United States (GenBank accession number MK059956), with nucleotide sequence identities ranging from 92.3 to 92.8%. Furthermore, the phylogenetic tree for the nucleotide sequences of ORF2 (Fig. 3C) for these three strains revealed that they clustered closely together among themselves, with nucleotide sequence identities ranging from 97.8 to 99.6%, and clustered closely together with reference strains of the classic HAstV1 genotype reported previously from the United Kingdom (GenBank accession number Z25771), China (GenBank accession numbers FJ755403 and KF211475), Hungary (GenBank accession number HQ398856), and the United States (GenBank accession numbers MN433703 and L23513), with nucleotide sequence identities ranging from 90.2 to 91.9%. Altogether, the data indicated that the nucleotide sequences of ORF1a, ORF1b, and ORF2 for CMH-N178-12, CMH-S059-15, and CMH-S062-15 were closely related to those of classic HAstV5, HAstV8, and HAstV1, respectively. The data suggest that CMH-N178-12, CMH-S059-15, and CMH-S062-15 are triple intergenotype recombinant strains of classic HAstV5, HAstV8, and HAstV1 genotypes within ORF1a, ORF1b, and ORF2, respectively.

### Analysis of recombination breakpoints.

**FIG 4:**
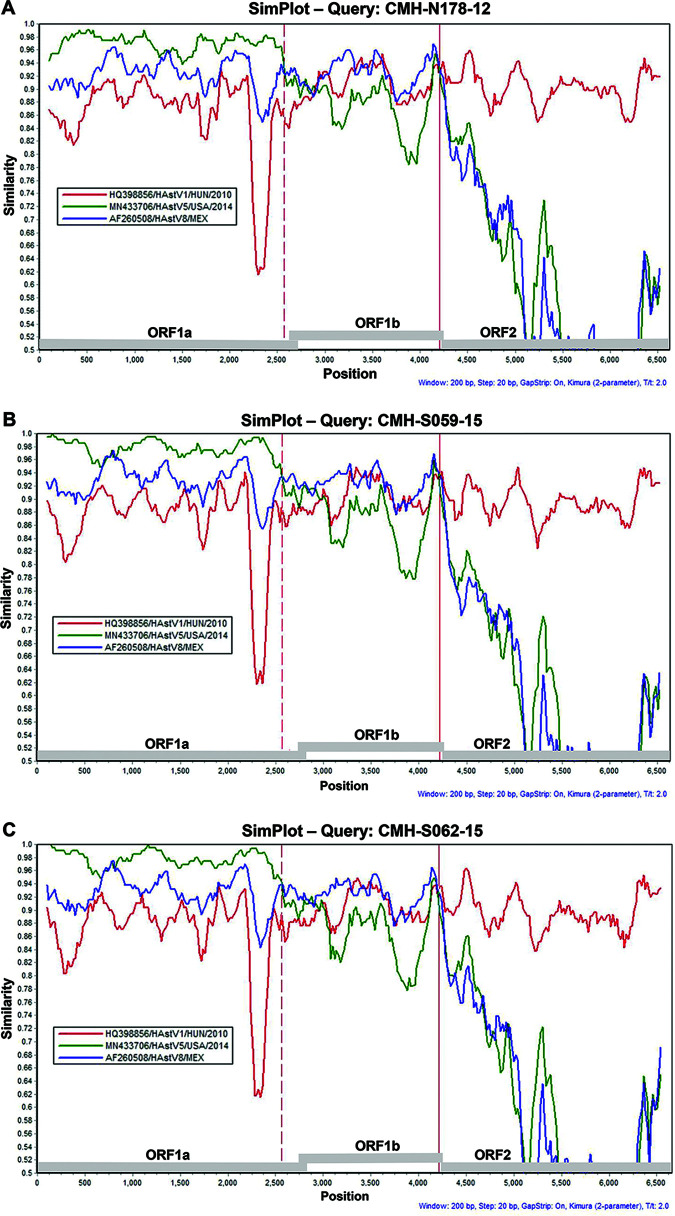
Similarity plot for AstV recombinant strains detected in Chiang Mai, Thailand. Similarity plots of nearly complete genome sequences of CMH-N178-12 (A), CMH-S059-15 (B), and CMH-S062-15 (C) are shown. The line intersect defines the predicted recombination site (vertical dashed lines in the ORF1a and solid lines in the ORF1b). The *x* axis represents the nucleotide position, and the *y* axis indicates the similarity between the query strain and the reference strains. The reference strains for HAstV1 (GenBank accession number HQ398856), HAstV5 (GenBank accession number MN433706), and HAstV8 (GenBank accession number AF260508) were used for SimPlot analysis.

In order to identify the recombination patterns and potential recombination breakpoints within the ORF1a, ORF1b, and ORF2 regions of the nearly full-length genomes of the putative recombinant strains, SimPlot software and the Recombination Detection Program (RDP) were used as tools for analysis, as shown in Fig. 4. A similarity plot generated by SimPlot based on 6,607 nucleotides of the nearly complete genome of the CMH-N178-12 strain as a query sequence, with HAstV1/HUN/2010 (GenBank accession number HQ398856), HAstV5/USA/2014 (GenBank accession number MN433706), and HAstV8/MEX (GenBank accession number AF260508) as the representative reference sequences for the HAstV1, HAstV5, and HAstV8 genotypes, respectively, is shown in Fig. 4A. The recombination breakpoints for CMH-N178-12 were located in the 3′-end regions of ORF1a and ORF1b, at nucleotide positions 2681 and 4357, respectively (Table 1). The recombination breakpoints for CMH-S059-15 (Fig. 4B) and CMH-S062-15 (Fig. 4C) were located at the same nucleotide positions within the 3′-end regions of ORF1a and ORF1b, at nucleotide positions 2612 and 4357 of ORF1a and ORF1b, respectively (Table 1). The nucleotide positions described in this study were based on the nucleotide positions of the full-length genome of the classic HAstV1 reference strain HAstV1/HUN/2010 (GenBank accession number HQ398856). The patient demographic features and the characteristics of the HAstV recombinant strains detected in the present study are shown in Table 1.

**TABLE 1:** Patient demographic features and characteristics of HAstV recombinant strains

Collection date	Strain name	Gender	Age (mo)	HAstV genotype^*a*^			Predicted recombination point (nucleotide position) by RDP analysis^*b*^	Recombination breakpoint in ORF^*b*^	*P* by RDP method
				ORF1a	ORF1b	ORF2			
11 December 2012	CMH-N178-12	M	NA^*c*^	HAstV5	HAstV8	HAstV1	2681	ORF1a	3.217 × 10^−20^
							4357	ORF1b	2.899 × 10^−34^
8 April 2015	CMH-S059-15	M	18	HAstV5	HAstV8	HAstV1	2612	ORF1a	2.203 × 10^−26^
							4357	ORF1b	1.593 × 10^−33^
8 April 2015	CMH-S062-15	F	17	HAstV5	HAstV8	HAstV1	2612	ORF1a	7.214 × 10^−27^
							4357	ORF1b	1.250 × 10^−33^

a The reference strains for HAstV1 (GenBank accession number HQ398856), HAstV5 (GenBank accession number MN433706), and HAstV8 (GenBank accession number AF260508) were used for SimPlot and RDP analyses. The recombination breakpoint location was based on the reference strain HAstV1 (GenBank accession number HQ398856).

b The ORF1a-ORF1b breakpoint was analyzed by RDP, comparing HAstV5 and HAstV8. The ORF1b-ORF2 breakpoint was analyzed by RDP, comparing HAstV8 and HAstV1.

c NA, not available.

## DISCUSSION

Recombination events and mutations are the major factors that play important roles in the genetic diversity and evolution of astroviruses (AstVs) and other RNA viruses (3, 4, 18). Recombination can interfere with the phylogenetic reconstruction of genome evolution and the calculation of the mutation accumulation rate of the viruses (19). Recombination is the exchange of genetic material between two genotypically different viruses, which leads to the generation of new virus progeny containing genetic material from both parents (20). This phenomenon occurs when two different viruses infect the same host cell and exchange genetic segments during replication. The exchange of genome fragments via homologous recombination is common in single-stranded RNA viruses. This event appears to occur at higher frequency in highly conserved genomic regions and between genetically related strains (18, 21, 22). HAstV is a single-stranded, positive-sense RNA virus, and its genome has undergone multiple recombination events, which had mainly occurred at the ORF1b-ORF2 (RdRp-capsid) junction region. The ORF1b-ORF2 overlapping region, which contains conserved sequences, is thought to act as a promoter for the transcription of subgenomic RNA, and this may increase the probability of homologous recombination (23).

Coinfection with different AstV genotypes within the same host can facilitate the occurrence of recombination and lead to the emergence of novel AstV strains (5, 24). Presumably, the emergence of a recombinant strain allows the new virus to adapt and successfully escape from the host immune system, eventually emerging as the predominant genotype to infect human populations (4). Previous research studies demonstrated that HAstV recombinant strains caused severe acute gastroenteritis as either sole infections or mixed infections with other bacterial, viral, or parasitic pathogens (14, 25). Another study reported that HAstV recombinant strains played a major role in causing diarrhea in children and adults in India. The authors demonstrated that the infection rate for HAstV recombinant strains was higher (84%) than that for single-specificity strains (16%) (15). Furthermore, Ulloa and Gutiérrez (26) described a recombinant strain between porcine AstV (PAstV) and HAstV in fecal samples obtained from piglets in Colombia. They showed a high prevalence of PAstV types that were closely related to HAstV in pigs. Together, these data imply the effects of recombinant virus on disease severity and the emergence of the predominant strains infecting humans and animals.

The ORF1b-ORF2 junction of the AstV genome is a hot spot at which recombination events frequently occur (6, 8, 9, 27, 28). In addition, recombination within the ORF1a gene has been reported (7, 15). A number of studies reported multiple patterns of HAstV intergenotype recombination of two regions, i.e., ORF1a-ORF2 and ORF1b-ORF2; however, triple recombinations within three regions of the HAstV genome, i.e., ORF1a, ORF1b, and ORF2, are rarely reported. To date, to the best of our knowledge, only two reports of triple recombinant strains of HAstV6/7-HAstV3-HAstV2 in the ORF1a-ORF1b-ORF2 regions have been documented (7, 10). In this study, we characterized the full-length genome sequences of three HAstV recombinant strains (CMH-N178-12, CMH-S059-15, and CMH-S062-15) detected in pediatric patients who had been admitted to the hospital with acute gastroenteritis. Phylogenetic analysis of the ORF1a (2,710 bp), ORF1b (1,540 bp), and ORF2 (2,350 bp) regions revealed that ORF1a, ORF1b, and ORF2 of these three strains belonged to the HAstV5, HAstV8, and HAstV1 genotypes, respectively. The present study provided new evidence of triple intergenotype recombinant strains of three different genotypes, HAstV5, HAstV8, and HAstV1, for the ORF1a, ORF1b, and ORF2 regions, respectively. Among the three recombinant strains, one strain (CMH-N178-12) was collected in 2012 whereas the other two strains (CMH-S059-15 and CMH-S062-15) were collected in 2015. It is interesting to point out that these three strains exhibited a potential recombination breakpoint at the same position in ORF1b (nucleotide position 4357) and two of them (CMH-S059 and CMH-S062-15) also carried the same recombination breakpoint in ORF1a at nucleotide position 2612. Two recombinant strains (CMH-S059 and CMH-S062-15) showed very close genetic relatedness to each other, with ORF1a, ORF1b, ORF2, and whole-genome nucleotide sequence identities of 99.9%, 99.9%, 99.6%, and 99.8%, respectively. High levels of identity between these sequences indicated that they were probably derived from the same parental strain. Recombination requires coinfection with different AstV strains in the same cell. Although coinfection cases with two or more HAstVs were not identified in the specimens analyzed in this study, the patients were from the same geographical area, where different HAstV genotypes, including HAstV1, HAstV2, HAstV3, HAstV4, HAstV5, and HAstV8, were cocirculating. Therefore, cocirculation of multiple HAstV strains in the same or nearby areas can provide the ideal setting for recombination to occur.

The ORF1a (29) and ORF1b regions contain sequences that are highly conserved among HAstV strains, whereas ORF2 is highly variable (30) and is commonly used as the basis for genotyping and phylogenetic analyses of viruses (31, 32). Generally, HAstV genotype assignments based on the ORF1a and ORF1b sequences are in agreement with those based on ORF2, except for genotyping of recombinant strains (32). A number of studies have identified HAstV recombinant strains by showing that either ORF1a (14, 15) or ORF1b (5, 7–10) genotypes are in disagreement with those for the ORF2 region. Identification of HAstV genotypes based on a short sequence of either ORF1a, ORF1b, or ORF2 alone may fail to detect the overall genetic diversity of circulating HAstV strains, while evidence for multiple intergenotype recombinations within the HAstV genome is being increasingly reported worldwide (7, 10) and was also clearly demonstrated in this study. In this regard, whole-genome sequence analysis would be of benefit not only for identifying multiple recombination breakpoints in the HAstV genome but also for providing information that leads to a better understanding of the genetic diversity and evolution of HAstV strains circulating in the region.

In conclusion, to the best of our knowledge, this is the first study to reveal full-length genome sequences of HAstV recombinant strains with a novel recombination pattern of ORF1a-ORF1b-ORF2 genotypes. This finding may be useful as a guideline for identifying other recombinant HAstV strains in other geographical regions and may provide a better understanding of their genetic diversity, as well as basic knowledge regarding virus evolution.

## MATERIALS AND METHODS

### HAstV strains.

A total of 92 archival HAstV strains detected in children <5 years of age who were hospitalized with acute gastroenteritis in Chiang Mai, Thailand, in 2011 to 2020 were included in this study. The RdRp genotypes of these 92 strains had been identified by ORF1b nucleotide sequence analysis in our previous studies (16, 17). Of these strains, 70 were classic HAstV genotypes (HAstV1, HAstV2, HAstV3, HAstV4, HAstV5, and HAstV8), 19 were novel HAstV-MLB genotypes (MLB1 and MLB2), and 3 were novel HAstV-VA2 genotypes.

### ORF1a nucleotide sequence analysis of HAstV strains.

In order to screen for a potential recombinant strain, the ORF1a genotype of these 92 HAstV strains was determined by ORF1a nucleotide sequence analysis. The viral RNA genomes of these 92 HAstV strains were extracted using a viral nucleic acid extraction kit II (Geneaid Biotech, Taiwan) according to the manufacturer’s protocol. The cDNA was synthesized from the viral RNA genome using a RevertAid first-strand cDNA synthesis kit (Thermo Fisher Scientific, USA). The ORF1a region was amplified by using the primer sets listed in Table 2. The amplicons were detected by 1.5% agarose gel electrophoresis and purified using a GenepHlow gel/PCR kit (Geneaid Biotech). The purified PCR products were sequenced by using the Applied Biosystems Sanger sequencing kit (Thermo Fisher Scientific) with an automatic genetic analyzer (Applied Biosystems) provided by Apical Scientific Sdn. Bhd. (Malaysia) (formerly known as First BASE Laboratories Sdn. Bhd.). The sequences obtained were analyzed to assign the genotype of the ORF1a region by comparison with those of the reference strains available in the GenBank database using the Basic Local Alignment Search Tool (BLAST) server (https://blast.ncbi.nlm.nih.gov/Blast.cgi) and phylogenetic analysis.

**TABLE 2:** Primers used for amplification of ORF1a of AstVs

Target	Primer name (direction)	Sequence (5′ to 3′)	Nucleotide positions^*a*^	PCR product size (bp)	Reference
Classic HAstV	MON340 (forward)	CGTCATTATTTGTTGTCATACT	1182–1203		32
	R1983 (reverse)	CGCTAACACTTTTCTGTTCTAG	1961–1983	802	This study
Novel HAstV-MLB1	SF0080 (forward)	AAGGATAGTGCTGGTAAAGTAGTTCAGA	1062–1089		
	SF0094 (reverse)	CAAGAGCCTTATCAACAACGTA	2268–2289	1,228	34
Novel HAstV-MLB2	SF0080-2 (forward)	AAGGATAACACTGGTAAGGTTGTTC	1062–1086		
	SF0094-2 (reverse)	CAAGGGCTTTATCTACCACAA	2269–2289	1,228	This study
Novel HAstV-VA	VA1067 (forward)	GCATGTTCATGAGTGACATTG	1067–1087		
	VA2102 (reverse)	TCTTTGTACTCTTCTTCAGTCCA	2080–2102	1,036	This study

a The primer locations for classic HAstV, novel HAstV-MLB1, novel HAstV-MLB2, and novel HAstV-VA strains were based on the nucleotide sequence positions of reference strains in GenBank (GenBank accession numbers KF211475, FJ222451, FJ742759, and GQ502193, respectively).

### Nearly complete genome sequence analysis of putative HAstV recombinant strains.

Nearly full-length genomes of putative HAstV recombinant strains were amplified by using the primer sets listed in Table 3. A total of 9 overlapping fragments encompassing a nearly full-length sequence of the HAstV genome for each HAstV strain were amplified, purified, and sequenced. The sequences obtained were assembled and manually edited to generate a nearly full-length viral genome by BioEdit v7.0 and MEGA11 software (33).

**TABLE 3:** Primers used for nearly complete genome amplification of HAstVs

Primer name	Sequence (5′ to 3′)	PCR product size (bp)	Reference
F30	CADGATGGCACWCGGTGAGCCAT		
R1451	CCAACGACATGTGCTGCTGTTACTAT	1,422	This study^*a*^
F1269	ATGGGAAGGTTGTGGCCACWGTACCAAC		
R 2091	CTACCCCTACCATGYTTGGTCTT	822	This study^*a*^
F1960	CACTAGAACAGAAAAGTGTTAGCG		
R 2844	CCTTGGTCTTCTGGGGCCCTTTGTAGT	884	35
F 2708	ATTATTGAAACAGCCATAAARAC		This study^*a*^
R 3543	GCCTTCCATTGGTGACCACCCACAT	835	35
F 3357	AGGAGACAAACCTGARGTACTTTGG		This study^*a*^
R 4335	CCTCCCCTCCAAATGCGATGGAGT	978	35
F4174	ACCTGAYYTTGAATCACTCCATGGGAAGC		
R 4714	CCAAAYTGAGTRCTCCCAGTAGC	540	This study^*a*^
F4562	TTGTCAATAARCAACTCAGGAAACARGG		35
R5399	ACCACCARCCTCCCTTDACHAGCCA	837	This study^*a*^
F5240	ATGAATGTWCCAGAGSVMAGCCATTTTG		
R5963	GCTDGTTABCTGGTGBTTRTT	723	This study^*a*^
F5755	AGGMCATKAYRMTGYKARRRTTG		
R6759	TCTAAACAGAGACAGAAAAGAAT	1,004	This study^*a*^

a These primers were modified from the report by El-Taweel et al. (35) based on all of the major classic HAstV genotype reference strains available in the GenBank database. The mixed bases in the degenerate primer are as follows: R = A/G, Y = C/T, M = A/C, K = G/T, S = C/G, W = A/T, H = A/C/T, B = C/G/T, V = A/C/G, D = A/G/T, and N = A/C/G/T.

### Phylogenetic and recombination analyses.

The phylogenetic trees for the ORF1a, ORF1b, and ORF2 genes were constructed by using MEGA11 software with the maximum likelihood method, and the best-fit evolutionary model for the data set was selected via the Tamura three-parameter model with 1,000 replicates. All of the prototype reference strains for the HAstV genotype and the global reference strains reported previously from several countries around the world whose whole-genome sequences were available in the GenBank database were selected, and the HAstV strains that were shown to be closely related to the HAstV strains detected in this study by using BLAST were a top priority to be included in the phylogenetic analysis. SimPlot v3.5.1 was used to analyze recombination events that occurred within the genome of AstV, and the recombination breakpoint was determined by using RDP v4.39. In addition, the homologous position of the recombination breakpoint was identified based on nucleotide sequence alignment of these strains together with other HAstV reference strains.

### Ethics approval.

The study was conducted with the approval of the ethical committee for human rights related to human experimentation, Faculty of Medicine, Chiang Mai University (study code: MIC-2557-02710; approval number: 2710/2014).

### Data availability.

The nearly full-length genome sequences of HAstV recombinant strains described in this study have been deposited in the GenBank database under the accession numbers ON979521 to ON979523. The nucleotide sequences of the partial ORF1b region of 92 AstV strains are available in the GenBank database under accession numbers MH325213 to MH325215, MH235217 to MH325266, and MZ327095 to MZ327133. The nucleotide sequences of the partial ORF1a region have been submitted to the GenBank database under accession numbers OQ543014 to OQ543105.
